# Supplementary material for: A Humanized Yeast Phenomic Model of Deoxycytidine Kinase to Predict Genetic Buffering of Nucleoside Analog Cytotoxicity
Source: Genes (Basel). 2019 Sep 30;10(10):770. doi: 10.3390/genes10100770 (PMC6826991; doi:10.3390/genes10100770)
Supplement: Supplementary file 1 [file genes-10-00770-s001.zip › Additional_Files/Additional_File7_GOTermHeatmaps.zip!/Additional_File7_GOTermHeatmaps/mitochondrial_matrix.pdf]

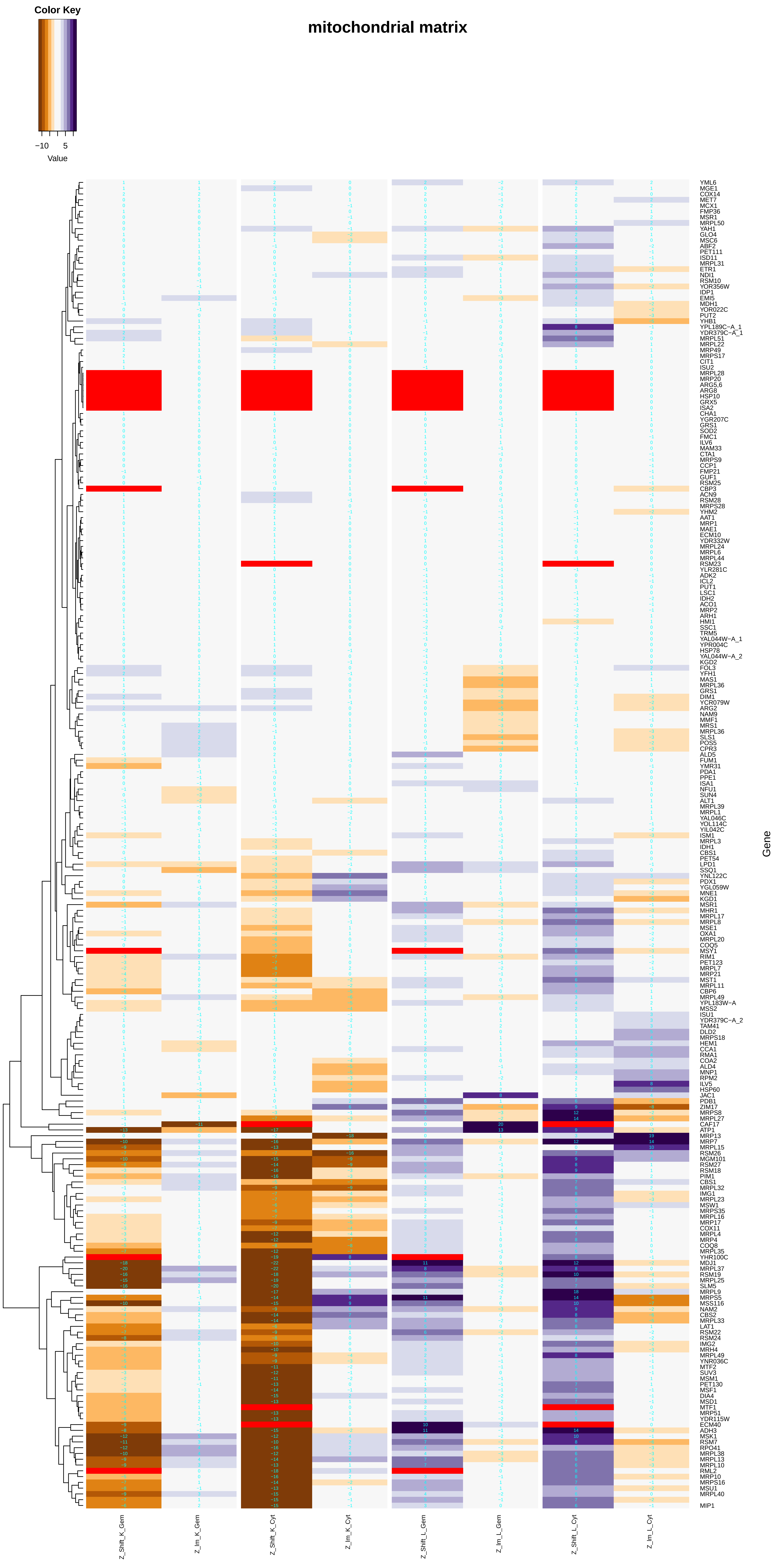
mitochondrial matrix
Color Key
−10
5
Value
Gene
YML6
MGE1
COX14
MET7
MCX1
FMP36
MSR1
MRPL50
YAH1
GLO4
MSC6
ABF2
PET111
ISD11
MRPL31
ETR1
NDI1
RSM10
YOR356W
IDP1
EMI5
MDH1
YOR022C
PUT2
YHB1
YPL189C−A_1
YDR379C−A_1
MRPL51
MRPL22
MRP49
MRPS17
CIT1
ISU2
MRPL28
MRPL20
ARG5,6
ARG8
HSP10
GRX5
ISA2
CHA1
YGR207C
GRS1
SOD2
FMC1
ILV6
MAM33
CTA1
MRPS9
CCP1
FMP21
GUF1
RSM25
CBP3
ACN9
RSM28
MRPS28
YHM2
AAT1
MRP1
MAE1
ECM10
YDR332W
MRPL24
MRPL6
MRPL44
RSM23
YLR281C
ADK2
ICL2
PUT1
LSC1
IDH2
ACO1
MRP2
ARH1
HMI1
SSC1
TRM5
YAL044W−A_1
YPR004C
HSP78
YAL044W−A_2
KGD2
FOL3
YFH1
MAS1
MRPL36
GRS1
DIM1
YCR079W
ARG2
NAM9
MMF1
MRS1
MRPL36
SLS1
POS5
CPR3
ALD5
FUM1
YMR31
PDA1
PPE1
ISA1
NFU1
SUN4
ALT1
MRPL39
MRPL1
YAL046C
YOL114C
YIL042C
ISM1
MRPL3
IDH1
CBS1
PET54
LPD1
SSQ1
YNL122C
PDX1
YGL059W
MNE1
KGD1
MSR1
MHR1
MRPL17
MRPL8
MSE1
OXA1
MRPL20
COQ5
MSY1
RIM1
PET123
MRPL7
MRP21
MST1
MRPL11
CBP6
MRPL49
YPL183W−A
MSS2
ISU1
YDR379C−A_2
TAM41
DLD2
MRPS18
HEM1
CCA1
RMA1
COA2
ALD4
MNP1
RPM2
ILV5
HSP60
JAC1
PDB1
ZIM17
MRPS8
MRPL27
CAF17
ATP1
MRP13
MRP7
MRPL15
RSM26
MGM101
RSM27
RSM18
PIM1
CBS1
MRPL32
IMG1
MRPL23
MSW1
MRPS35
MRPL16
MRP17
COX11
MRPL4
MRP4
COQ8
MRPL35
YHR100C
MDJ1
MRPL37
RSM19
MRPL25
SLM5
MRPL9
MRPS5
MSS116
NAM2
CBS2
MRPL33
LAT1
RSM22
RSM24
IMG2
MRH4
MRPL49
YNR036C
MTF2
SUV3
MSM1
PET130
MSF1
DIA4
MSD1
MTF1
MRP51
YDR115W
ECM40
ADH3
MSK1
RSM7
RPO41
MRPL38
MRPL13
MRPL10
RML2
MRP10
MRPS16
MSU1
MRPL40
MIP1
Z_Shift_K_Gem
Z_Im_K_Gem
Z_Shift_K_Cyt
Z_Im_K_Cyt
Z_Shift_L_Gem
Z_Im_L_Gem
Z_Shift_L_Cyt
Z_Im_L_Cyt
